# Along signal paths: an empirical gene set approach exploiting pathway topology

**DOI:** 10.1093/nar/gks866

**Published:** 2012-09-21

**Authors:** Paolo Martini, Gabriele Sales, M. Sofia Massa, Monica Chiogna, Chiara Romualdi

**Affiliations:** ^1^CRIBI Biotechnology Center, ^2^Department of Biology, University of Padova, via U. Bassi 58/B, 35121 Padova, Italy, ^3^Department of Statistics, University of Oxford, 1 South Parks Road, Oxford, OX1 3TG, UK and ^4^Department of Statistical Science, University of Padova, via C. Battisti 241, Padova, Italy

## Abstract

Gene set analysis using biological pathways has become a widely used statistical approach for gene expression analysis. A biological pathway can be represented through a graph where genes and their interactions are, respectively, nodes and edges of the graph. From a biological point of view only some portions of a pathway are expected to be altered; however, few methods using pathway topology have been proposed and none of them tries to identify the signal paths, within a pathway, mostly involved in the biological problem. Here, we present a novel algorithm for pathway analysis clipper, that tries to fill in this gap. clipper implements a two-step empirical approach based on the exploitation of graph decomposition into a junction tree to reconstruct the most relevant signal path. In the first step clipper selects significant pathways according to statistical tests on the means and the concentration matrices of the graphs derived from pathway topologies. Then, it identifies within these pathways the signal paths having the greatest association with a specific phenotype. We test our approach on simulated and two real expression datasets. Our results demonstrate the efficacy of clipper in the identification of signal transduction paths totally coherent with the biological problem.

## INTRODUCTION

Recently much attention has been directed toward the study of gene sets in the context of microarray data analysis (hereafter GSA). A microarray experiment typically provides a list of differentially expressed genes (DEGs) (1,2) that represent the starting point of a highly challenging process of result interpretation. The grouping of genes into functionally related entities is of great help for interpreting the results. In this context, statistical methods for the identification of groups of functionally related genes with moderate, but coordinated, expression changes are fundamental to help biologists in the process of results comprehension.

Several GSA tests, both univariate and multivariate, have been recently developed (3–7). GSA methods can be divided into two broad categories: (i) methods based on enrichment analysis performed on a list of genes selected through a gene-level test; and (ii) methods based on global and multivariate approaches that define a model on the whole gene set (8). In general these two approaches are based on two fundamentally different null hypotheses: the first type hypothesizes the same level of association of a gene set with the given phenotype as the complement of the gene set (say, Q1). The second type only considers the genes within a gene set and hypothesizes that there is no gene in the gene set associated with the phenotype (say, Q2) (9). Goeman and Buhlmann (5) termed these approaches *competitive* and *self-contained*, respectively. The main drawbacks with *competitive* methods are (i) the assumption that genes are independent; and (ii) the use of a cut-off threshold for the selection of DEGs. In this way, many genes with moderate but meaningful expression changes are discarded by the strict cut-off value, which leads to a reduction in statistical power. On the other hand, global and multivariate approaches relax the assumption of independence among genes belonging to the same gene sets and identify moderate, but coordinated, expression changes that cannot be detected by the previous approach without depending from any arbitrary cut-offs.

In general, the *a priori* definition of gene sets is obtained from Gene Ontology (GO) (10) information or from biological pathways; while genes belonging to a GO category do not have any explicit connections among them (apart from being involved in the same function), genes in the same pathway are structured in a network with explicit biological interactions. Almost all of the *self-contained* approaches, when applied to biological pathways, use merely the list of genes belonging to a pathway, and therefore, although effective, miss the relevant topological information contained.

In the last years, little effort has been done to consider the topological information within the *self-contained* GSA methods. The seminal paper by Draghici *et al.* (4) proposed an interesting approach (called Impact Analysis, SPIA (11)) attempting to capture several aspects of the data: the fold change of DEGs, the pathway enrichment and the topology of signaling pathways. In particular, SPIA enhances the impact of a pathway if the DEGs tend to lie near its entry points. Recently, Isci *et al.* (6) proposed a Bayesian pathway analysis that models each biological pathway as a Bayesian network and considers the degree to which the model fits the observed experimental data. Both approaches test the whole pathway without providing the user with the portions of the pathway that are effectively associated with the phenotype. This is an essential information especially when the pathway is large.

To this end, Laurent *et al.* (12) developed a graph-structured two-sample test of means for problems in which the distribution shift is assumed to be smooth on a given graph and devised branch and bound algorithms to systematically apply their test to the subgraphs of a large graph, without enumerating and testing these subgraphs one-by-one. Alternatively, Massa *et al.* (13) introduced an innovative approach based on Gaussian graphical models that tests both differences in mean and in covariance matrices between two experimental conditions. In particular, the graphical models context is useful to decompose the overall graph (obtained from the pathway) into smaller parts (cliques), that can be explored and tested in detail.

An alternative approach was proposed by Emmert-Streib (7) that proposes to infer the undirected dependency graphs representing pathways. Briefly, given two groups, Emmert-Streib (7) infers the dependency structure of genes belonging to the same GO group using Pearson correlation and partial Pearson correlation independently on both groups, and then tests the similarity of the inferred graphs using a graph edit distance and a permutational approach.

In this work, we take the starting point that pathways are the best representation of biological experimentally validated knowledge of a specific process. In fact, the annotation of a biological pathway is the result of an extensive effort of hundreds of researchers that manually codify their experimental knowledge about a specific biological process into a graphical representation. Therefore, we decide to consider the topology of the pathway as fixed.

Following Massa *et al.* (13), we propose an empirical two-step method, called clipper hereafter, for the identification of significant signal transduction paths within significantly altered pathways. In particular: (i) we generalize the approach of Massa *et al.* (13) to the case of *P* ≫ *n* (with *P* number of genes/variables and *n* number of samples/replicates), using shrinkage and a graphical lasso penalty estimators of the covariance matrices; and (ii) by exploiting the structure of a junction tree derived from an initial graph, we propose a procedure to highlight the portions, called signal paths, of a pathway mostly correlated with the phenotype.

We test our approach on simulated and real expression datasets of completely different biological problems (cancer and muscle disorders). The obtained results provide evidence of the success of our approach in the detection of altered pathways and, more importantly, in the identification of novel signal paths. We believe that clipper could become an important tool for gene expression data interpretation.

## MATERIALS AND METHODS

To implement topology-based GSA using microarray data, we need first to convert pathways into gene networks, i.e. into a graphical structure in which a node represents a simple element like a gene/protein (14). In fact, whereas pathway nodes might consist of multiple entities such as protein complexes, gene family members and chemical compounds, microarrays measure each single element of complexes and gene family separately. Here, we used graphite (14), a Bioconductor package addressing these issues. In general, graphite takes pathway information from four different databases (Biocarta; KEGG, (15); NCI/Nature Pathway Interaction Database, (16); Reactome, (17)) and this information is interpreted and opportunely coded by following specific biologically driven rules. Specifically, given a pathway structure, graphite converts it into a gene–gene network. We refer to the manual of the package for more information on the conversion.

Pathways may be cyclic or acyclic. The number of pathways with cycles is dependent either on the structure of the graph or on the number of genes in the array, but fortunately is quite small. Given that the graphical inference methods assume to have an acyclic graph we preventively eliminate self-loops and solve cycles removing the weakest edge of the cycle based on expression data (with minimum expression profile correlation between nodes) (see also (18)).

Then, an acyclic gene network can be read as a Directed Acyclic Graph (DAG). Most inference methods for a DAG convert the network to an undirected cycle-free graph. Such conversion might require some or all of the following steps: moralization, triangulation, clique identification and junction tree construction. Briefly, moralization inserts an undirected edge between two nodes that have a child in common and then eliminates directions on the edges; triangulation inserts edges in the moralized graph so that in the moralized graph all cycles of size ≥4 have chords, where a chord is defined as an edge connecting two non-adjacent nodes of a cycle; clique identification identifies the cliques of the triangulated graph, i.e. the complete subgraphs having all their vertices joined by an edge; junction tree construction builds a new hyper-tree having cliques as nodes and satisfying the *running intersection property* according to which, for any cliques 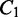 and 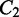 in the tree, every clique on the path connecting 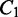 and 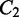 contains 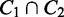. As an example, consider the pathway Chronic myeloid leukemia (CML) from KEGG database, see Supplementary Figure S1.

## STEP 1: TESTING THE WHOLE PATHWAY

In specific conditions, the strength of molecular interactions within a pathway could be altered, making the pathway a dynamic entity. It is therefore reasonable to test its dynamic perturbation by statistically testing equality of concentration matrices and mean vectors. Here, we assume to have two classes of samples (say cases and controls) and we suggest to model the data in the two experimental conditions with two graphical Gaussian models with the same undirected graph *G*:
(1)
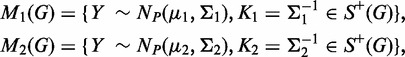

where *P* is the number of genes (vertices of the graph), 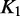 and 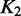 are the concentration matrices (inverse of the covariance matrices) of the two models and 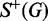 is the set of symmetric positive definite matrices with null elements corresponding to the missing edges of *G*. Here, *G* is the graph obtained after transforming the network obtained from graphite first into a DAG, and, then, into its moral graph.

In Massa *et al.* (13), two tests were proposed, one for the comparison of the strength of the links between genes in the two experimental conditions and another one to test the differential expression of the pathway. In the first case, the hypothesis to be tested is 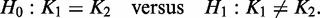 Testing the differential expression of the pathway is achieved by checking equality of means, i.e. 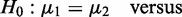 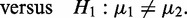 Such test has a different structure according to whether the two graphical Gaussian models 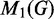 and 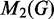 are homoschedastic, i.e. they have the same covariance matrix, or not.

Once the graph *G* is known, the null elements in the concentration matrices are identified. On the contrary, 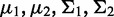 are not known and need to be estimated from the data. Here, 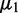 and 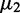 are estimated with the corresponding sample means. The maximum-likelihood estimates of 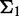 and 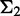 can be obtained by using the Iterative Proportional Scaling algorithm (IPS, see (19, p.134)) and by taking the sample covariance matrices as starting values. The IPS guarantees that the estimated matrices belong to 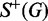. In this case, a necessary condition for the existence of the maximum-likelihood estimate is that the number of samples is greater than the cardinality, i.e. the number of nodes of the largest clique (19, p. 133), a setting that is easily missed in case of gene expression data (a typical microarray experiment does not exceed the few tens of samples per class, and with the advent of deep-sequencing technology, this dimension is further reduced to few units). In Supplementary Figure S2, we report the distribution of the cardinality of the largest clique *per* pathway in four different databases. It is worth noting that there are several pathways with clique cardinality of several tens of nodes that would not be processed by the standard IPS algorithm.

To estimate the covariance matrix in such circumstances, clipper applies a shrinking procedure in the estimation of the sample covariance matrices. Apart from increased efficiency, the shrunken estimates have the additional advantage of being always positive definite and well conditioned. Here, we use a James–Stein-type shrinkage estimator, as implemented in corpcor R package (20,21).

The shrunken estimates are passed on to the IPS algorithm. The use of a shrinkage estimator, however, precludes the use of the asymptotic distribution of the 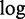-likelihood ratio test which, in standard settings, has a 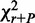 distribution under the homoschedasticity hypothesis, where *r* is the number of edges and *P* the number of nodes of the graph. Here, we will use a permutational approach on the samples.

Even if the IPS algorithm implemented in qpgraph (22) is one of most computationally efficient, in some cases (very large and complex pathways) it is highly computationally demanding (e.g. for diverse pathways the IPS algorithms takes even several days to converge) and sometimes it has problems of convergence. Therefore, with clipper, we have also investigated the possibility of computing the maximum-likelihood estimate of the covariance matrices using the approach of Friedman *et al.* (23), implemented in the R package glasso, where we have specified the indices of entries of the inverse covariance matrix to be constrained to zero and set the regularization parameter equal to zero.

As expected, the estimates of the covariance matrices obtained by glasso with no regularization and with the IPS algorithm are the same. However, we do not find significant improvement in the computational efficiency and both approaches show the same average computation time.

To compare portions of the pathways, with the aim of identifying subgroups of genes which appear to drive differences (deregulations) of the entire structure, clipper performs the above described tests on each single clique. To this end, the moral graph is first triangulated (if needed). As the cliques are complete connected subgraphs, the IPS algorithm is not required to estimate covariances.

## STEP 2: IDENTIFICATION OF RELEVANT SIGNAL PATHS

Using the structure of the junction tree as a backbone, clipper empirically identifies the portions of the tree mostly associated to the phenotype. For each pathway and the corresponding moralized graph, our approach is based on three main steps: (i) construction of the junction tree; (ii) identification of paths and corresponding sub-paths; and (iii) computation of the relevance of the sub-paths as specified below.

We define a *path* as the path connecting the root clique with a leaf clique [identified by maximum cardinality search (mcs) algorithm]. For each clique along the paths, we consider the *P*-value of the test on homoschedasticity as weight *w* of the clique. From now on, such quantities will loose their probability interpretation although they will still reflect the importance of the clique difference between the experimental conditions. A weight will be considered to be meaningful if it is <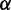. In our analysis we set 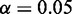; however, different cut-offs can be used. On each path, we select the portions of the path composed by consecutive meaningful cliques containing at most one non-meaningful clique. Such portions define the so called *sub-paths*.

**Figure 1.**
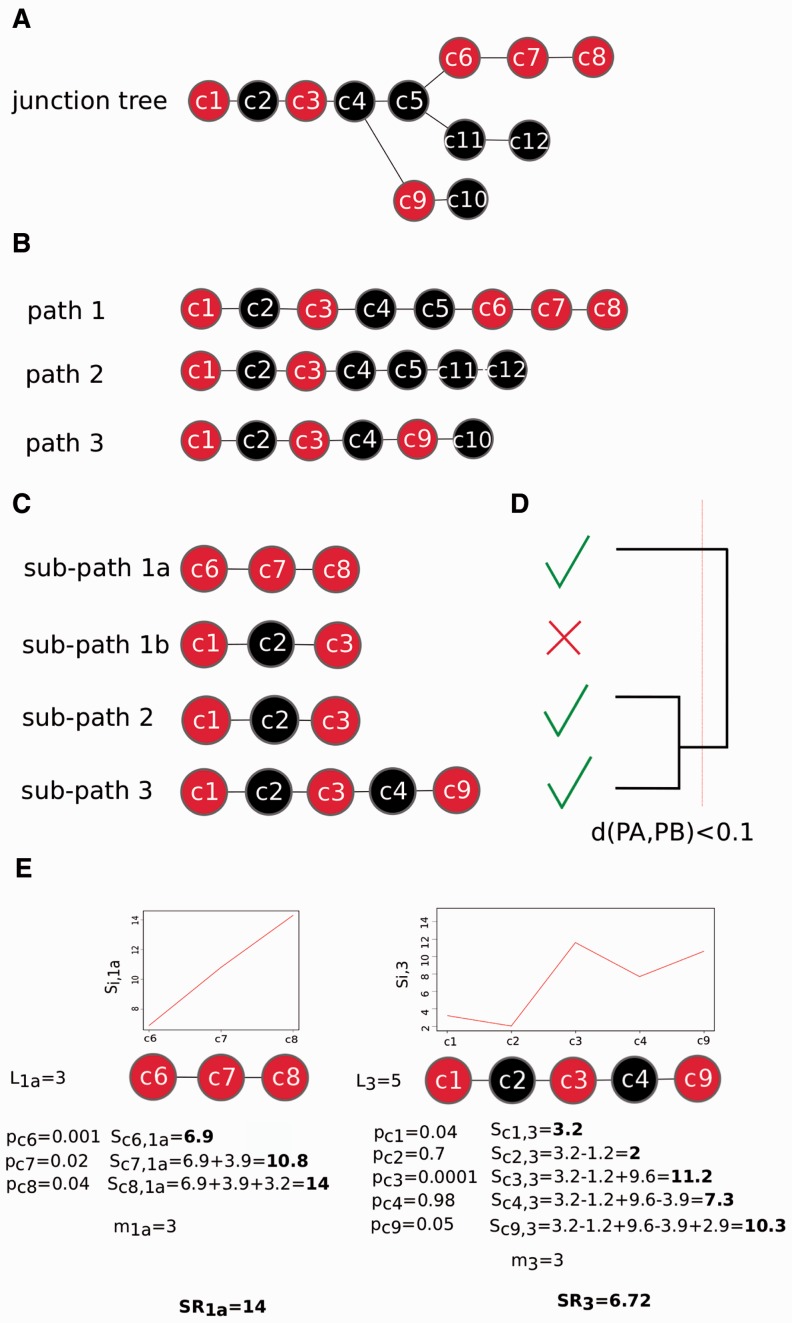
Toy example of step 2 clipper approach. Panel **A**, the construction of the junction tree with significant cliques in red. Panel **B**, identification of the *paths* in the tree. Panel **C**, identification of all the *sub-paths* within each *path*. Panel **D**, selection of the best *sub-path* for each *path* and cluster analysis for sub-path collapse. Panel **E** Final *sub-path* selected.

An example of the above described steps is given in Figure 1. Panel A represents a junction tree with root clique *c*1 and three leaf cliques, i.e. *c*8, *c*10 and *c*12. Meaningful cliques are highlighted in red. Panel B represents the three paths derived from the junction tree. Panel C reports the four sub-paths which can be extracted from the path.

The relevance of each sub-path is computed as follows. Let 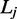 be the length of sub-path *j*, with *j* = 1, … , *J*. Given the weight 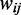 of each clique *i* in the sub-path *j*, 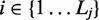, the relevance is calculated according to Equation (2). Respecting the ordering of the cliques in the sub-path, for each clique *i* in sub-path *j*, we compute the quantity
(2)
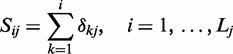

where 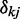 is defined as
(3)
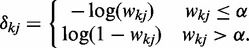

Then, the relevance 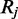 of sub-path *j* is defined to be the maximum of 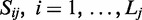. To compare the relevance of sub-paths of different lengths, we introduce the standardized relevance 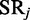

(4)
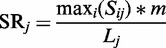

where *m* is the position of the 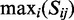 along the sub-path *j*. Finally, for each path, the sub-path with the maximum 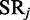 is selected as its relevant signal path. At the end of this procedure, a relevant signal path is identified for each path.

clipper results consist of a number of relevant signal paths. In most of the cases, paths and sub-paths are highly overlapping (see, e.g., sub-paths 1b, 2 and 3 in Figure 1). Thus, clipper implements a pruning procedure using a cluster analysis approach. We define the dissimilarity measure between sub-paths *A* and *B*, *d*(*A, B*), as
(5)
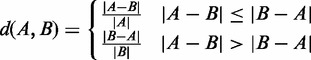

where A and B are the sets of genes composing sub-paths *A* and *B*, 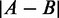 is the cardinality of sets difference and 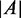 is the cardinality of the set *A* (similarly are defined 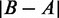 and 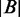). We perform a cluster analysis and collapse sub-paths with 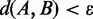 (taking the sub-path with the highest relevance). For our analysis, we set 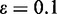; however, clipper allows the selection of a different threshold. For a numerical example, see panel E of Figure 1.

## RESULTS

### Rationale

Different experimental conditions are usually compared in terms of their gene expression mean differences. In the univariate case, if a gene increases or decreases significantly its mean expression in one condition with respect to the other, it is said to be differentially expressed and it is assumed to be involved in the biological process under study. It is easy to generalize the previous concept to the multivariate setting; if a gene set changes significantly its multivariate mean expression in one condition with respect to the other, it is said to be differentially expressed. However, the difference in mean expression levels does not necessarily result in a change of the interaction strength among genes. In this case, we will have pathways with significant altered mean expression levels but unaltered biological interactions.

On the contrary, if transcripts abundances ratios are altered, we expect a significant alteration not only of their mean expression levels, but also of the strength of their connections, resulting in pathways with completely corrupted functionality. Therefore, to look for pathways strongly involved in a biological process, we should look at pathways with both mean and variance significantly altered.

clipper is based on a two-step approach: (i) it selects pathways with both covariance matrices and means significantly different between experimental conditions; and (ii) on such pathways, it identifies the sub-paths mostly associated to the phenotype. clipper is freely available as an R package at http://romualdi.bio.unipd.it/ in Software section.

In this section, we provide (i) a simulation study to test the specificity of our approach; and (ii) an application of clipper on two real datasets along with a comparison with GSEA (3) (non-topological method), SPIA (11) and BPA (6) (topological methods). Differently from BPA, SPIA requires a list of DEGs. Here, we used empirical Bayes test (1) to identify DEGs (implemented in limma Bioconductor package). On real datasets, clipper step 2 will be applied to one of the pathways identified in step 1.

### Simulation

As some paths may be declared relevant by clipper step 2 simply as a consequence of type I errors in clipper step 1, we developed a simulation study. For 10 000 runs, we generated two samples, one for each condition, from the same graphical model 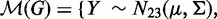

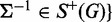
 and tested equality of concentration matrices and mean vectors for the whole pathway and all the cliques. Under this scenario, at the nominal level 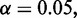 we expected: (i) for each test, a number of rejections around 5%; (ii) a scattered location along the junction tree of the statistically significant cliques. (ii) implies that the length of significant paths identified by clipper step 1 should be rarely (about 5%) longer than 1. Results shown in Supplementary Table S1 demonstrate that our procedures have an excellent control of type I error in step 1 and very appreciably respond to expectations in step 2, even with exceptionally low sample sizes.

### Application: ALL dataset

The dataset we use for this comparison was published by Chiaretti *et al.* (24) and characterizes gene expression signatures in acute lymphocytic leukemia (ALL) cells associated with known genotypic abnormalities in adult patients. Several distinct genetic mechanisms lead to ALL malignant transformations deriving from distinct lymphoid precursor cells that have been committed to either T-lineage or B-lineage differentiation. Chromosome translocations and molecular rearrangements are common events in B-lineage ALL and reflect distinct mechanisms of transformation. The relative frequencies of specific molecular rearrangements differ in children and adults with B-lineage ALL. The BCR breakpoint cluster region and the c-abl oncogene 1 (BCR/ABL) gene rearrangement occurs in about 25% of cases in adult ALL, and much less frequently in pediatric ALL.

Data are available at the Bioconductor site (http://www.bioconductor.org/help/publications/2003/Chiaretti/chiaretti2/). Expression values, appropriately normalized according to robust multiarray analysis (rma) and quantile normalization, derived from Affymetrix single channel technology, consist of 37 observations from one experimental condition (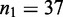, BCR; presence of BCR/ABL gene rearrangement) and 41 observations from another experimental condition (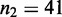, NEG; absence of rearrangement). Probes platform have been annotate using EntrezGene custom CDF version 14 (25).

**Step 1 results**

Given the presence of the BCR/ABL chimera, we expect that all the pathways including BCR and/or ABL1 will be impacted. The KEGG pathways found to be significantly involved (Bonferroni adjusted *P*-value 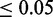) in the difference between translocation positive and negative patients by clipper step 1 analysis are reported in Table 1. Firstly, it is worth noting that with an adjusted *P*-value 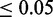 clipper identifies as significantly deregulated almost all (7 out of 9 *P*-value = 3.279616e–06) pathways including BCR and/or ABL genes (in red Table 1). On the contrary, GSEA, SPIA and BPA did not find any significantly altered pathways using Bonferroni adjusted *P*-value 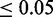. However, if uncorrected *P*-value 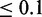 is considered, SPIA and GSEA identify 2 out of 9 (*P*-value = 0.18) pathways, including either ABL and/or BCR genes (Table 1), while BPA identifies only one.

**Table 1.** KEGG significant pathways of according to the test on the means and the test on the concentration matrices

ID	Pathway name	Adj. *P*-values test 1^a^	Adj. *P*-values test 2^b^	SPIA^$^	BPA^$^	GSEA^$^
1	Adherens junction	0	0.00e + 00			Yes
2	Cell cycle	0	0.00e + 00		Yes	
3	Dilated cardiomyopathy	0	0.00e + 00			
4	Measles	0	0.00e + 00			
5	Prostate cancer	0	0.00e + 00			Yes
6	Regulation of actin cytoskeleton	0	0.00e + 00			Yes
7	Vascular smooth muscle contraction	0	0.00e + 00			
8	Wnt signaling pathway	0	0.00e + 00	Yes		Yes
9	Natural killer cell-mediated cytotoxicity	0	5.76e − 14			
10	Bacterial invasion of epithelial cells	0	7.68e − 14			
11	Melanogenesis	0	1.54e − 13			Yes
12	Tight junction	0	8.34e − 12			Yes
13	Toll-like receptor signaling pathway	0	1.68e − 10	Yes		
14	Viral myocarditis	0	2.63e − 10	Yes		
15	Axon guidance	0	1.31e − 09			
16	Basal cell carcinoma	0	5.90e − 09	Yes		Yes
17	Insulin signaling pathway	0	1.39e − 08	Yes		
18	Acute myeloid leukemia	0	2.44e − 08			
19	Neurotrophin signaling pathway	0	6.69e − 08			
20	Glycolysis/gluconeogenesis	0	8.00e − 08			
21	Shigellosis	0	2.04e − 07			
22	TGF-beta signaling pathway	0	3.71e − 07			
23	Leukocyte transendothelial migration	0	9.40e − 07			Yes
24	T cell receptor signaling pathway	0	3.37e − 06			
25	Chronic myeloid leukemia	0	4.40e − 06			
26	Leishmaniasis	0	1.65e − 05			
27	Fructose and mannose metabolism	0	1.78e − 05			
28	Systemic lupus erythematosus	0	6.32e − 05			
29	Pyruvate metabolism	0	1.71e − 04			
30	Fc gamma R-mediated phagocytosis	0	6.34e − 03			Yes
31	RIG-I-like receptor signaling pathway	0	7.03e − 03	Yes		
32	Pathogenic *Escherichia coli* infection	0	8.13e − 03	Yes		Yes
33	B cell receptor signaling pathway	0	2.77e − 02			

In red those pathways including BCR and/or ABL genes, in blue those pathways coherent with experimental evidences.

^a^Test on the mean with Bonferroni correction.

^b^Test on the concentration matrices with Bonferroni correction.

^$^SPIA, BPA and GSEA results using raw *P*-value 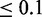.

Moreover, most of the other pathways identified by clipper are strongly coherent with experimental findings on BCR/ABL mechanism. In fact, many signaling proteins have been shown to interact with BCR/ABL through various functional domains/motifs (e.g. GRB2, CRKL, CRK, SHC, 3BP2, ABL-interacting protein 1 and 2, and CRK-associated substrate (CAS)), and/or to become phosphorylated in BCRABL-expressing cells (e.g. CRKL, CRK, SHC, GAB2, CBL, CAS, the p85 subunit of PI3K, FES, paxillin and talin). These proteins, in turn, activate a range of signaling pathways identified by clipper (in blue Table 1) that activate proteins such as RAS, PI3K,A KT, JNK, SRC family kinases, protein and lipid phosphatases, and their respective downstream targets, as well as transcription factors such as the STATs, nuclear factor-kB and MYC. Most of these findings were observed from experiments *in vitro* systems, or from studies of the properties of cells derived from leukaemia patients with particular stages of disease (26).

**Step 2 results**

**Figure 2.**
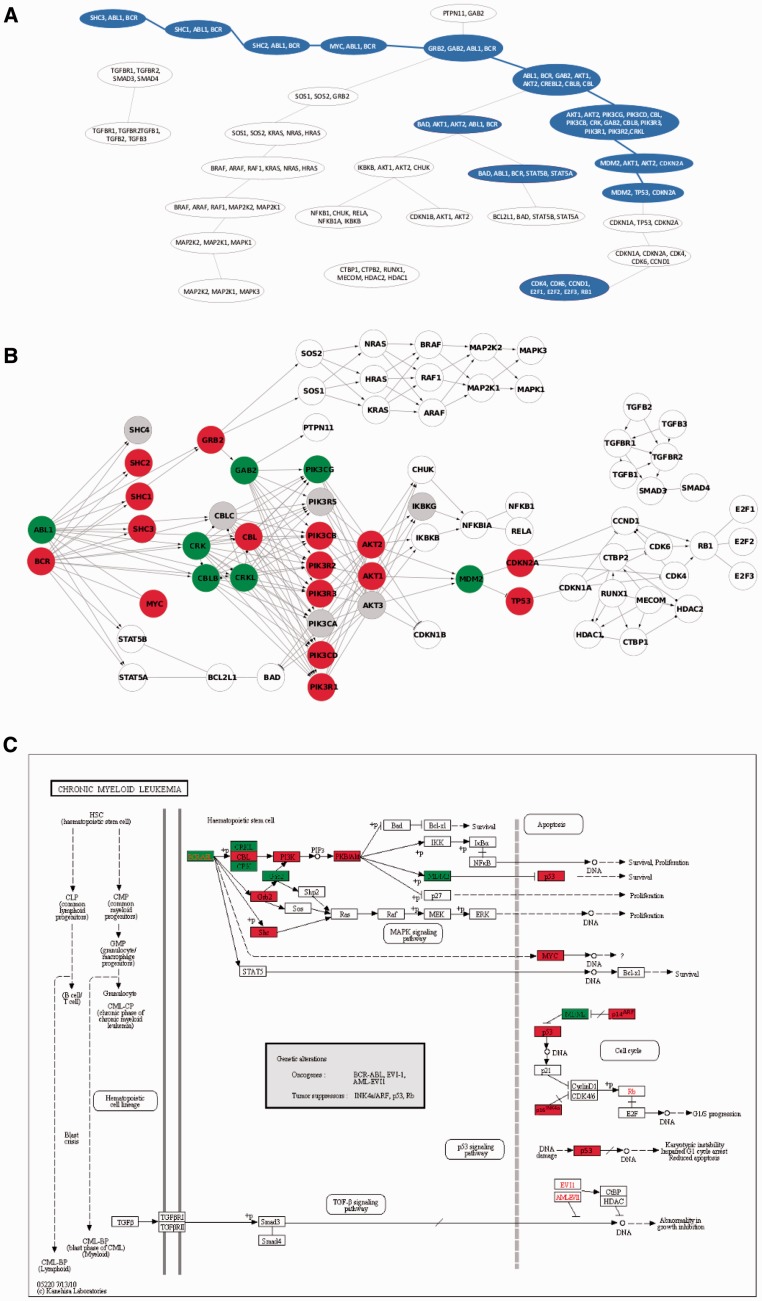
clipper results on chronic myeloid leukaemia (CML) KEGG pathway. Panel **A**, junction tree with significant cliques in blue. The highest scored *sub-path* is highlighted with blue border. Panel **B**, CML pathway with genes belonging to significant cliques in red or green according to their expression mean differences (translocation positive versus negative patients). Panel **C**, the original KEGG CML layout with complexes belonging to the sub-path identified colored according to their expression.

Focusing on CML pathway that contains exactly BCR/ABL fusion gene, clipper identifies a sub-path that fits perfectly with experimental findings. In particular, the highest scoring sub-path is that one starting from BCR/ABL toward the oncogene TP53 (Figure 2). It is known, in fact, that the BCR/ABL fusion protein in CML cells, promotes the accumulation of p53 and that, in contrast to the activation of p53 by c-Abl, its oncogenic form, BCR/ABL, counteracts the growth inhibitory activities of p53 by modulating the p53-MDMD2 loop. Thus, it appears that by modulating the p53-MDMD2 loop, c-Abl and its oncogenic forms critically determine the type and extent of the cellular response to DNA damage (27).

It is worth noting that the signal path obtained by clipper would have not been identified using just the list of DEGs belonging to this pathway. Only ABL1 and NFKBIA, in fact, are identified by empirical Bayes test (1) as differentially expressed with 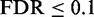.

### Application: LGMDs dataset

Limb girdle muscular dystrophies (LGMDs) are a group of muscular diseases with heterogeneous clinically and genetically features. Globally, they present progressive muscle weakness caused by progressive muscle waste combined with an increase of muscle connective tissue. We analyse a dataset containing 10 LGMD type 2A (LGMD2A) and 10 type 2B (LGMD2B) samples (28).

LGMD2A is caused by a mutation in the gene calpain3 (29) that codes for a cysteine protease that cleaves cytosckeletal and myofibrillar proteins and serves to maintain proper functions and structure of the sarcomere (30). LGMD2B is caused by a mutation in the gene dysferlin that codes for a sarcolemma protein involved in membrane repair and muscle regeneration (31). Together with desmoyokin (AHNAK), dysferlin forms the dysferlin protein complex involved in the maintenance of the sarcolemma integrity (32). AHNAK is also a substrate of calpain3, and after the cleavage AHNK is not able to bind dysferlin anymore confirming the mutual influence that calpain3 and dysferlin protein exert each other (32). Thus, we expect few molecular differences between these pathologies.

**Step 1 results**

In the analysis for LGMDs, we used Reactome and KEGG databases stored in graphite. Firstly, we identify the involvement of Apoptosis (e.g. pathways 1 and 4 in Table 2). In case of stress signals, proapoptotic BCL-2 family proteins are activated and subsequently interact with and inactivate antiapoptotic BCL-2 proteins. This interaction leads to the destabilization of the mitochondrial membrane and release of apoptotic factors that reduce muscle cell survival in LGMD2A (33). Moreover, clipper results help in formulating novel hypothesis on this case study. Specifically, we found many pathways referred to MAPK signaling (e.g. pathways 2 and 5 in Table 2). Our results seem in agreement with (34) that recently showed the role of MAPK signaling pathway in the LMNA-associated degenerative process and the similarity of the regulatory processes between LGMD2A, LGMD2B and LMNA-associated muscular dystrophy regardless of the causative gene.

**Step 2 results**

**Figure 3.**
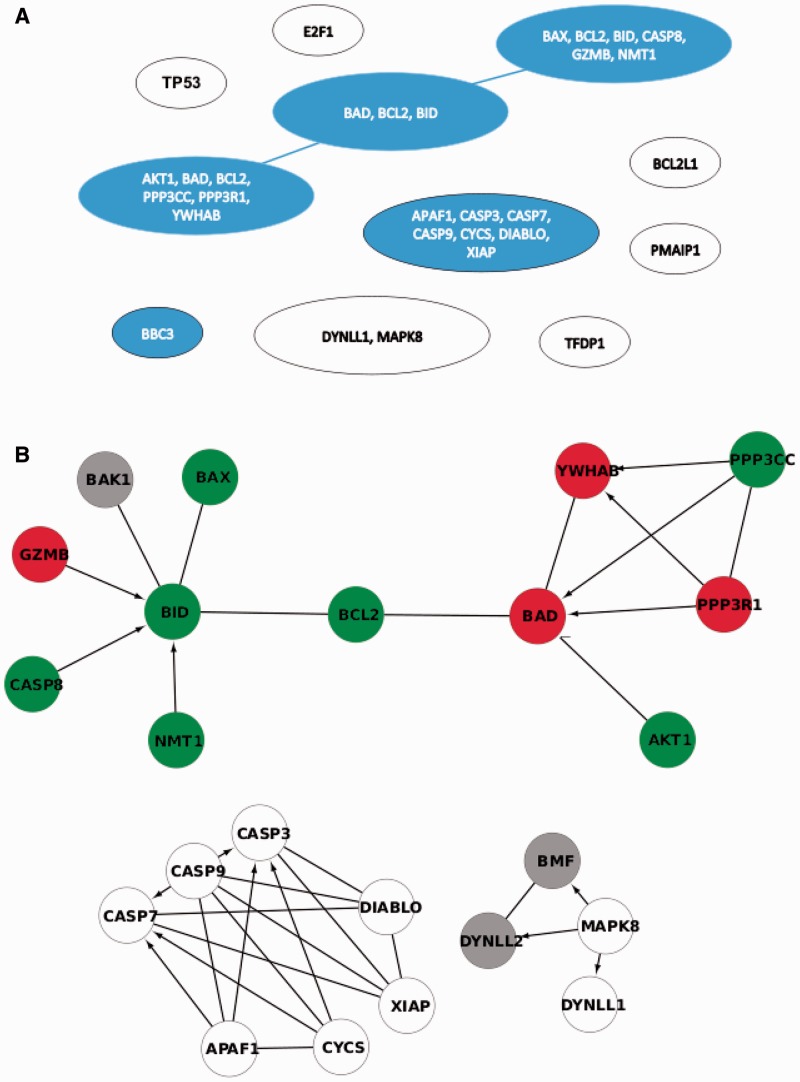
Intrinsic pathway of apoptosis. Panel **A**, junction tree with significant clique in blue. The highest scored *sub-path* is highlighted with blue border. Panel **B**, native pathway with genes belonging to significant cliques in red or green according to their expression mean differences (LGMD2A versus LGMD2B).

With the step 2 of clipper analysis, we are able to reach an even deeper level of accurateness. We focused on Intrinsic pathway of apoptosis. Figure 3 shows that the signal sub-path identified by clipper include BAX, BID, BCL2 and BAD that play a central role in leading to apoptosis.

**Table 2.** List of significant KEGG and Reactome pathways according to the test on the means and the test on the concentration matrices

	Pathway name	Adj. *P*-values test 1^a^	Adj *P*-values test 2^b^
1	KEGG: RIG-I-like receptor signaling pathway	0	5.68e − 13
2	Reactome: GRB2:SOS provides linkage to MAPK signaling for integrins	0	3.22e − 13
3	Reactome: DCC-mediated attractive signaling	0	8.50e − 09
4	Reactome: Intrinsic pathway for apoptosis	0	1.07e − 06
5	Reactome: p130Cas linkage to MAPK signaling for integrins	0	1.37e − 06
6	Reactome: TRAIL signaling	0	1.50e − 02
7	Reactome: signal regulatory protein (SIRP) family interactions	0	2.00e − 02
8	Reactome: activation of BH3-only proteins	1	2.16e − 03

BPA cannot be performed on Reactome database and GSEA does not identify significantly deregulated pathways, neither with Bonferroni adjusted *P*-values nor with nominal *P*-values.

^a^Test on the mean with Bonferroni correction.

^b^Test on the concentration matrices with Bonferroni correction.

## CONCLUSIONS

Here, we present clipper, a novel two-step empirical method for pathway analysis able to dissect the complexity of a pathway identifying the portions mostly associated to the biological process studied.

Our empirical approach is fundamentally different from previous ones for two reasons. We take into account not only expression changes but also differences in transcript concentrations, allowing the identification of pathways with their functionality completely corrupted. We are able to go to the finest details of the pathway structure, identifying the signal transduction path that is the principal cause of the pathway deregulation.

clipper efficacy has been validated on two expression datasets of completely different biological problems (cancer and muscle disorders). In both cases, we obtained interesting results strongly coherent with experimental findings available in literature. Moreover, our results demonstrate the utility of clipper not only in the result comprehension but also in driving the experimenter in formulating new hypothesis. We therefore believe that clipper would become an important tool for gene expression data interpretation.

## SUPPLEMENTARY DATA

Supplementary Data are available at NAR Online: Supplementary Table 1 and Supplementary Figures 1 and 2.

## FUNDING

Funding for open access charge: University of Padova [CPDA119031 to C.R. and M.C.].

*Conflict of interest statement*. None declared.****

## Supplementary Material

Supplementary Data

## APPENDIX

### Gaussian graphical models

We report here a concise review of Gaussian Graphical Models theory. A graph *G* is a pair , where *V* is a finite set of vertices and the set of edges  is the set of ordered pairs of distinct vertices. If both  and , the edge (*u, v*) is said to be undirected. If  but , the edge (*v, u*) is said to be directed.

A DAG is a directed graph without cycles. Given a DAG, a moral graph is the undirected graph obtained from the DAG by adding undirected edges between all pairs of vertices that have a child in common (if they are not already present) and then by rendering all edges undirected.

If *G* is undirected, then a subgraph is *complete* if all its vertices are joined by an edge. Any *complete* subgraph is a *clique*. A maximal complete subgraph (with respect to ) is a maximal *clique*. In a graphical models context only maximal cliques are relevant in estimation problems and therefore we will always use the term clique with the meaning of maximal clique.

A triple (*A, B, C*) of disjoint subsets of *V* of an undirected graph *G* is a decomposition of *G* if , *C* is a complete subset of *V* and *C* separates *A* and *B*. An undirected graph is *decomposable* if either it is complete or it possesses a proper decomposition (*A, B, C*) such that both subgraphs  and  are decomposable.

A *triangulated graph* (or *chordal graph*) is an undirected graph with the property that every cycle of length  has two non-consecutive vertices that are adjacent. An important result is that an undirected graph is decomposable if and only if it is triangulated (19, p. 9). If a graph is not triangulated, it is possible to add extra edges so that the resulting graph is triangulated. It is well known that the problem of obtaining an optimal triangulation (i.e. finding the smallest number of edges to be added) is NP-hard and therefore we rely on the heuristic algorithm developed in the R package gRbase, implemented in the function triangulate.

A *junction tree of cliques* for a graph *G* is a tree having the cliques of *G* as nodes and satisfying the *running intersection property* according to which, for any cliques  and  in the tree, every clique on the path connecting  and  contains . Decomposability is a necessary and sufficient condition for the existence of a junction tree. We build a junction tree by finding a running intersection property ordering of the cliques via the maximum cardinality search algorithm (mcs, implemented in the rip function of the gRbase R package).

A Gaussian graphical model with dependence graph *G* = (*V, E*) where  can be defined as the multivariate normal distribution   where  is the set of symmetric positive definite matrices with null elements corresponding to the missing edges of *G*.
