# Supplementary material for: Along signal paths: an empirical gene set approach exploiting pathway topology
Source: Nucleic Acids Res. 2012 Sep 21;41(1):e19. doi: 10.1093/nar/gks866 (PMC3592432; doi:10.1093/nar/gks866)
Supplement: Supplementary Data [file supp_41_1_e19__index.html]

Along signal paths: an empirical gene set approach exploiting pathway topology — Along signal paths: an empirical gene set approach exploiting pathway topology — Supplementary Data 

# Along signal paths: an empirical gene set approach exploiting pathway topology

## Supplementary Data

files

**Files in this Data Supplement:**

- Supplementary Data - pdf file
