# Supplementary material for: Along signal paths: an empirical gene set approach exploiting pathway topology
Source: Nucleic Acids Res. 2012 Sep 21;41(1):e19. doi: 10.1093/nar/gks866 (PMC3592432; doi:10.1093/nar/gks866)
Supplement: Supplementary Data [file supp_gks866_nar-01883-met-n-2012-File001.pdf]

Along signal paths: an empirical gene set approach exploiting pathway topology
Supplementary Information

Paolo Martini, Gabriele Sales, Sofia Massa, Monica Chiogna
Chiara Romualdi[1]

[1]To whom correspondence should be addressed. Tel: +39 049 8277401; Fax: +39 049 8276159; Email: chiara.romualdi@unipd.it

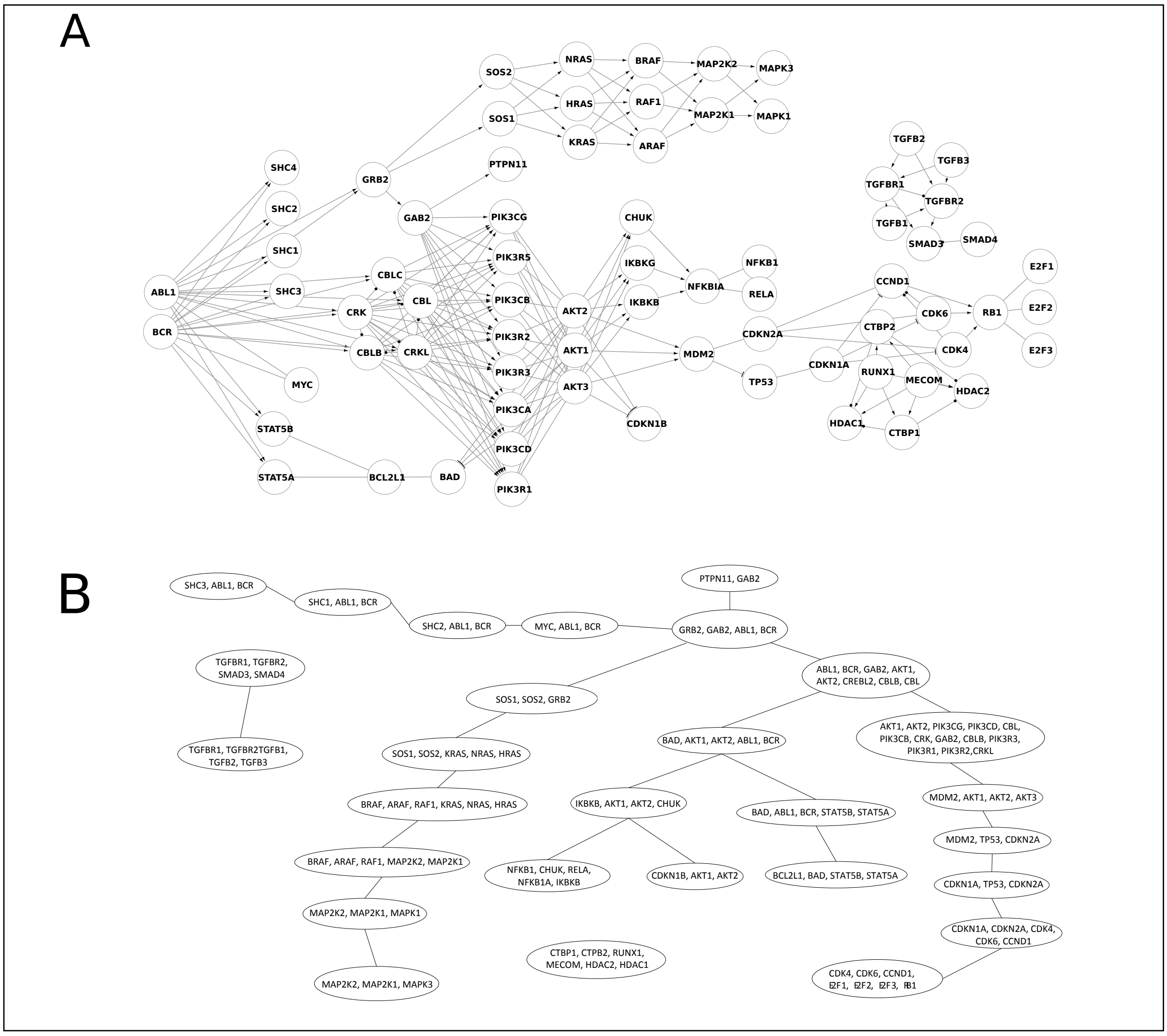

Figure 1: Chronic myeloid leukemia pathway. Panel A. Original pathway structure obtained by `graphite`. Panel B. Junction tree structure obtained by maximum cardinality search algorithm.

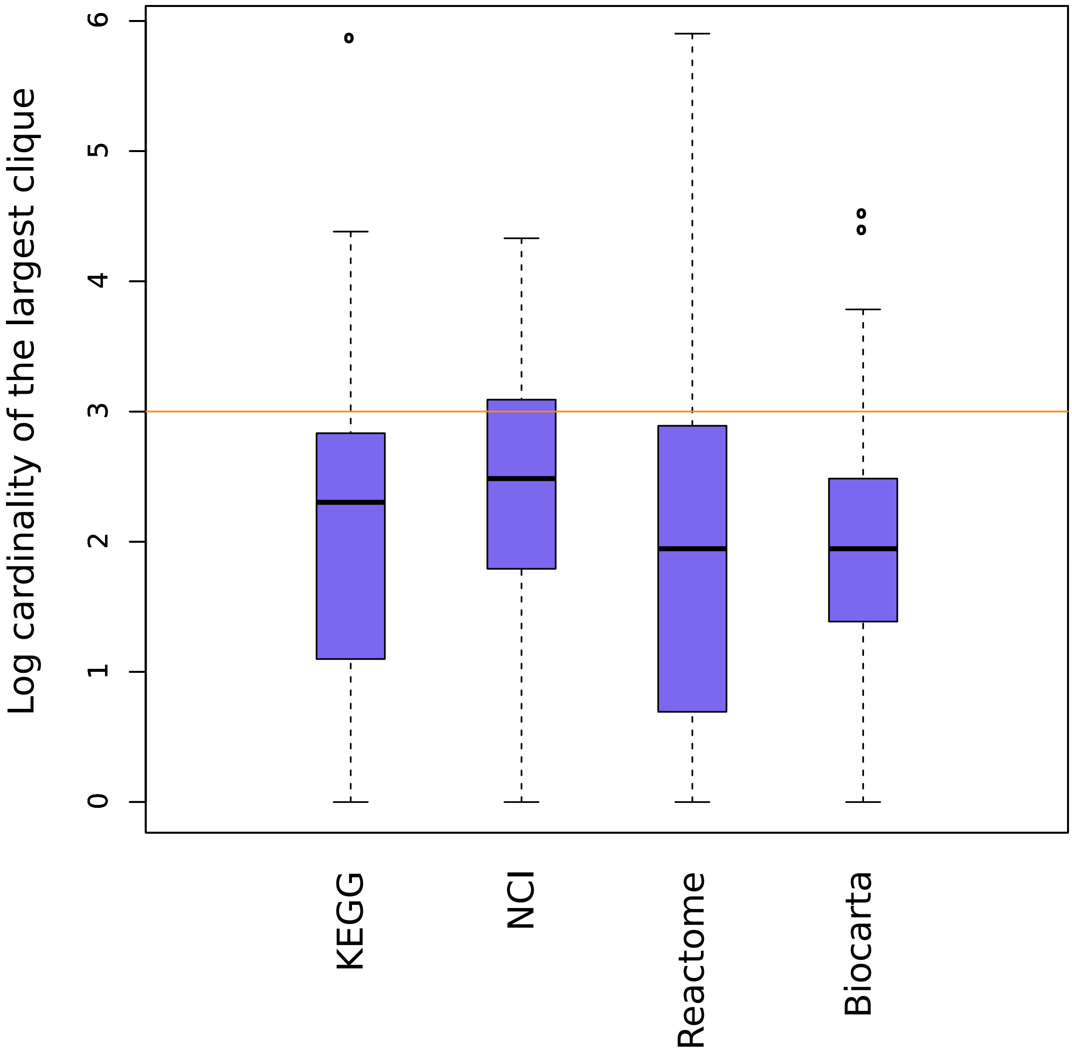

Figure 2: Distribution of the log-cardinality (logarithm of the number of nodes composing a clique) of the largest clique of the pathways for the four databases. The orange line corresponds to a clique cardinality of 20 ($ln(20) \approx 3$).

Table 1: Simulation results. Given a graph with 23 nodes and 24 edges, for 10000 runs we simulated two datasets, one for each condition. At each run, the hypotheses $H_0 : K_1 = K_2 \quad \text{vs} \quad H_1 : K_1 \neq K_2$ and $H_0 : \mu_1 = \mu_2 \quad \text{vs} \quad H_1 : \mu_1 \neq \mu_2$ have been tested. For each null hypothesis, the table reports the proportion of rejections, along with the proportion of relevant paths with length greater than one (a single clique).

Sample size	% of significant tests on variances	% of significant tests on means	% of Path length greater than 1
$N = 100$	6%	5%	0%
$N = 80$	6%	5%	5%
$N = 50$	5%	5%	9%
$N = 20$	6%	6%	10%
$N = 10$	6%	5%	10%
